# RECRUITMENT AND RETENTION STRATEGIES FOR BLACK AMERICAN DEMENTIA CAREGIVER PEER SUPPORT

**DOI:** 10.1093/geroni/igae098.0964

**Published:** 2024-12-31

**Authors:** Karen Moss, Kathy Wright, Mary Beth Happ, Abraham Brody, Celia Wills, Seuli Bose Brill, Karen Bullock

**Affiliations:** The Ohio State University, Columbus, Ohio, United States; The Ohio State University, Columbus, Ohio, United States; The Ohio State University, Columbus, Ohio, United States; HIGN, New York University Rory Meyers College of Nursing, New York, New York, United States; The Ohio State University, Columbus, Ohio, United States; The Ohio State University, Columbus, Ohio, United States; Boston College, Chestnut Hill, Massachusetts, United States

## Abstract

Black American caregivers of older adults living with dementia are at high risk for caregiving-related physical, emotional, spiritual, and psychosocial challenges. Culturally responsive interventions are needed to improve caregiving outcomes. Peer mentorship, a relationship-centered person-to-person approach may support caregiving mastery via culturally-tailored, flexible, non-judgmental peer support, including storytelling about caregiving experiences. The purpose of this presentation is to describe the recruitment and retention processes used for a pilot feasibility/acceptability study to test a co-created virtual intervention, called Peer Support for Caregivers of Black Americans Living with Dementia (Pair 2 Care). Community engagement strategies were used in Pair 2 Care such as: 1) use of co-design process to develop and improve the intervention, including community advisory board engagement, 2) individual and community-based organization relationship building over time, 3) principal investigator and research team in-person and virtual attendance at community health and caregiving events, 4) principal investigator contributions to community-based education programs, 5) reconnecting with previous research participants, 6) cultural preparation of research team members, and, 7) frequent contact with enrolled participants. A total of N=26 former caregivers (mentors) and current caregivers (mentees) were enrolled, mentors trained, and 1:1 dyads paired within 10 weeks for six months of peer support. Currently, retention is at 100% and early feedback from dyads is favorable. Recruitment and retention strategies outlined for this pilot study assisted the research team with successful study execution of an innovative culturally-tailored caregiver support intervention intended to build caregiving mastery and contribute to health equity by improving caregiver health outcomes.

